# A fault-tolerant method for HLA typing with PacBio data

**DOI:** 10.1186/1471-2105-15-296

**Published:** 2014-09-03

**Authors:** Chia-Jung Chang, Pei-Lung Chen, Wei-Shiung Yang, Kun-Mao Chao

**Affiliations:** Department of Computer Science and Information Engineering, National Taiwan University, No.1, Sec.4, Roosevelt Road, Taipei, 10617 Taiwan; Departments of Medical Genetics and Internal Medicine, National Taiwan University Hospital, No. 8, Chung Shan S. Road, Taipei, 10041 Taiwan; Graduate Institute of Clinical Medicine, National Taiwan University College of Medicine, No. 1, Sec. 1, Jen Ai Road, Taipei, 10051 Taiwan; Research Center for Developmental Biology and Regenerative Medicine, National Taiwan University, No.1, Sec.4, Roosevelt Road, Taipei, 10617 Taiwan; Graduate Institute of Medical Genomics and Proteomics, National Taiwan University, No.1, Sec.4, Roosevelt Road, Taipei, 10617 Taiwan; Graduate Institute of Biomedical Electronics and Bioinformatics, National Taiwan University, No.1, Sec.4, Roosevelt Road, Taipei, 10617 Taiwan

**Keywords:** HLA typing, NGS, PacBio

## Abstract

**Background:**

Human leukocyte antigen (HLA) genes are critical genes involved in important biomedical aspects, including organ transplantation, autoimmune diseases and infectious diseases. The gene family contains the most polymorphic genes in humans and the difference between two alleles is only a single base pair substitution in many cases. The next generation sequencing (NGS) technologies could be used for high throughput HLA typing but in silico methods are still needed to correctly assign the alleles of a sample. Computer scientists have developed such methods for various NGS platforms, such as Illumina, Roche 454 and Ion Torrent, based on the characteristics of the reads they generate. However, the method for PacBio reads was less addressed, probably owing to its high error rates. The PacBio system has the longest read length among available NGS platforms, and therefore is the only platform capable of having exon 2 and exon 3 of HLA genes on the same read to unequivocally solve the ambiguity problem caused by the “phasing” issue.

**Results:**

We proposed a new method BayesTyping1 to assign HLA alleles for PacBio circular consensus sequencing reads using Bayes’ theorem. The method was applied to simulated data of the three loci HLA-A, HLA-B and HLA-DRB1. The experimental results showed its capability to tolerate the disturbance of sequencing errors and external noise reads.

**Conclusions:**

The BayesTyping1 method could overcome the problems of HLA typing using PacBio reads, which mostly arise from sequencing errors of PacBio reads and the divergence of HLA genes, to some extent.

**Electronic supplementary material:**

The online version of this article (doi:10.1186/1471-2105-15-296) contains supplementary material, which is available to authorized users.

## Background

Human leukocyte antigen (HLA) system contains a set of genes that encode for major histocompatibility complex (MHC) in humans. The main function of MHC molecules is to mediate interactions between antigen-presenting cells, various lymphocytes and other body cells; therefore, malfunctions of HLA may associate with certain disorders in the immune system, for example, drug hypersensitivity reactions [1] and some autoimmune diseases, e.g., type 1 diabetes and systemic lupus erythematosus [2]. HLA also plays an important role in transplantation of organs or stem cells [3, 4] and is associated with infectious diseases such as HIV [5].

There are 10,533 HLA alleles in the IMGT/HLA Database [6] and the number is still increasing. The HLA genes are the most polymorphic genes in humans and the difference between two alleles is often only a single base pair substitution. There are two main classes of HLA genes. The class I HLA genes (HLA-A, -B, and -C) each encodes a glycoprotein chain in association with the monomorphic molecule *β*2-microglobulin on the cell surface of most somatic cells, and the class II HLA genes (HLA-DP, -DQ and -DR) each encodes an *α* or a *β* glycoprotein chain associated as heterodimers on the cell surface of antigen-presenting cells [7]. The exon 2 and exon 3 sequence of class I HLA genes and the exon 2 sequence of class II HLA genes form the critical peptide-binding groove responsible for the specificity of peptide recognition and binding [7].

It has been shown that high-resolution HLA matching improves survival rates of marrow transplantation [8]. Therefore, to identify the alleles of a sample, it is better to use DNA-based methods instead of serological approaches [9]. In addition, with the advance of the next generation sequencing (NGS) technologies, HLA typing by NGS seems to be a promising approach for HLA sequencing and allele assignment owing to its efficiency and cost effectiveness. [10] reviewed the latest approaches of template preparation, sequencing platforms and data-analysis for HLA typing by NGS. It was showed that the four major NGS platforms, Roche GS 454 FLX, Illumina MiSeq/HiSeq, PGM Ion Torrent and Pacific Biosciences SMRT (PacBio), were all capable of producing sequences suitable for the resolution of HLA genotyping.

However, among the four platforms, HLA typing by PacBio was the least addressed. For example, the module **HLA Typing** in the software **Omixon Target** from Omixon only works with Illumina, Roche 454 and Ion Torrent. The software **NGSengine** from GenDx is platform independent but is optimized for Roche 454, Ion Torrent, MiSeq. It might be due to the high error rate of PacBio (about 15-20%), which makes it more difficult to genotype polymorphic regions such as HLA. Moreover, to sequence multiple samples simultaneously, sequences could be labelled with barcodes for identification of samples [11]. With a higher error rate, there are more barcode-calling errors and reads are more likely identified as of wrong samples. To our knowledge, the troublesome issue related to wrong barcode assignment in PacBio HLA typing has not been addressed previously.

**Figure 1:**
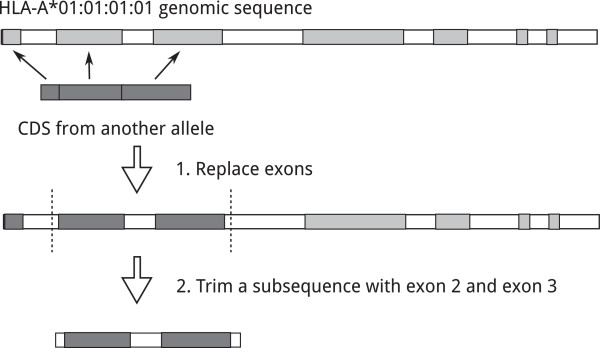
**Target sequences preparation.** We simulated target sequences for alleles that have only coding sequences in the IGMT/HLA Database. The exons of the reference genomic sequence were replaced by the coding sequence of each allele.

Despite the high raw error rate, the PacBio system actually has two very unique advantages for HLA genotyping. First, the PacBio system has the longest read length among available NGS platforms. According to the public documents from PacBio and the real-world data we received from the PacBio machine (personal communication with PacBio representatives), for data generated with P4-C2 chemistry, the average read length is about 5.5 Kb and the number of reads is about 50 K per SMRT cell. Although the class I HLA genes contain seven or eight exons as illustrated in Figure 1, their genotypes are determined by numerous (mostly) single nucleotide substitutions scattered/patched in both exon 2 and exon 3 (the trimmed regions in Figure 1). If the genetic variants in exon 2 and exon 3 are derived from different reads, then the correct phasing of the two exon 2 sequences and two exon 3 sequences from the same individual needs to be predicted by some computational algorithm, which inevitably causes “ambiguity” [7, 12]. PacBio is the only platform capable of having exon 2 and exon 3 of HLA genes on the same read to unequivocally solve the ambiguity problem. Second, although the raw error rate of PacBio is the highest among available NGS platforms, a unique advantage of PacBio is that the errors occur randomly without a systemic error pattern. Therefore, with sufficient coverage and appropriate error-correction techniques, the final assemble error rate can be one of the lowest among all the NGS platforms [13].

There are two main types of PacBio reads: continuous long read (CLR) and circular consensus sequencing read (CCS). (For a short enough insert, the PacBio system is capable of multi-pass sequencing the raw read and generates a consensus sequence with higher accuracy, i.e., CCS). Both types of reads can be used for targeted sequencing, which is to sequence specific areas of interest within the genome, (e.g. regions within the HLA genes in our application). The characteristics of CLR and CCS reads in the early days for targeted sequencing were previously summarized in [14] and we only excerpt a part in Table 1.

**Table 1:** **Characteristics of CLR and CCS reads for targeted sequencing applications excerpted from** [14]

	CLR	CCS
Read Accuracy	85-90%	≥98*%* (3 pass)
Maximum Mean Readlength	2 Kb	1 Kb
#Reads	100 K	15 K

#Reads here stands for the average number of usable reads per SMRT cell for 1 Kb insert.

To solve the challenging “ambiguity” related to genotyping of class I HLA genes, the targeted sequences have to cover the whole region of exon 2 and exon 3 (and also intron 2 in between the two exons), which is about 1 Kb, and the read lengths of both types fit the requirement. Both types of reads are also proved to be effective in detecting genomic variants [14]. However, in order to save cost for diagnosis applications of HLA typing, it is better to apply the barcode multiplexing technology [11]. With a high error rate, the barcodes of CLR reads are much more likely to be mis-mapped and the reads are more likely identified as of wrong samples. Therefore, we choose CCS reads as the target of our method.

One possibility to genotype HLA is to use assembly methods such as CAP3 and PCAP [15, 16] to recover the two genomic sequences of a sample and compare them with the alleles. However, there are still problems using CCS reads. First, the number of reads for each amplicon of each sample is not high (depending on the number of barcodes used). Take the experiment in [11] for example. There were 2,352 distinct sequence products (49 amplicons × 48 barcode pairs). When the insert size is 1 Kb, the average number of reads for each distinct product was only 6.38. Second, the error rate of CCS is still too high to distinguish two HLA alleles having only little base-pair difference. Third, there are still barcode-calling errors for CCS reads (data not shown), which could induce noise reads and increase the obstacles of HLA typing.

To address these problems, here we propose a method using Bayes’ theorem. Given a few CCS reads generated from the target sequence (of the regions containing exon 2 and exon 3 for class I HLA genes or of the regions containing exon 2 for class II genes) of a sample, our method is able to correctly assign the pair of alleles of the sample. We simulated the alleles for each sample and the CCS reads generated according to the alleles. Different levels of reads from wrong samples were added to disturb the experiments. The experimental results showed that our method can stand for a high percentage of noise reads.

## Methods

### Simulation

The simulation follows the setting of the multiplexing targeted sequencing technology [11]. In each run, there are multiple samples with multiple amplicons sequenced simultaneously. We assume that the reads have been grouped by their samples and amplicons (loci of HLA) and the reads might be identified as of wrong samples due to barcode-calling errors.

#### Alleles of the samples

The alleles of the samples were assigned following the distribution of the Taiwan Minnan population [17]. We obtained the frequencies of alleles from the allele frequency net database [18, 19]. For the alleles with zero frequency, we gave them 0.1% frequencies of appearance. In their study, only HLA-A, B and DRB1 are involved, so we only simulated HLA on these three loci. The table of frequencies for the three loci can be found in Additional file 1: Table S1. When implementing our methods, the frequencies were normalized to make the summation of each loci equal 1.

Linkage disequilibrium was not concerned, which means alleles on different loci are assigned independently. In reality, given the probability density function of the pairs of alleles in a population, we can adjust our methods by setting *p*(*a*_*i*_,*a*_*j*_) in Equation (1) accordingly. In addition, since the frequencies were censused for alleles with 2-digit resolution, the alleles of higher resolution were selected with uniform distribution. To observe the impacts of homozygous samples on genotyping, 30% of the samples have two identical alleles.

#### Target sequences of the alleles

The HLA sequences were downloaded from the IGMT/HLA Database Release 3.15.0 [20]. Since there are only CDSs instead of genomic sequences for most alleles in the database, we created the genomic sequences and corresponding target sequences of our own.

We illustrated the creation of the target sequences in Figure 1. First, for each locus, a reference genomic sequence was selected and the positions of its exons (the light grey blocks) were detected by aligning its CDS to its reference genomic sequence and we have the long rectangle intercepted with only the light grey block in Figure 1. Then, the genomic sequence of each allele was created by replacing the exons of the reference genomic sequence with the exon sequences of the allele (the continuous dark grep blocks), which were obtained from the CDS alignment file *locus*_nuc.txt downloaded from the HLA database. Now we have the long rectangle intercepted by some dark grey blocks (the exons from the selected allele) and other light grey blocks (the exons from the reference allele that the selected allele misses). The missing nucleotides (represented as * in *locus*_nuc.txt) are replaced with nucleotides of another sequenced allele in the same positions. Most sequence-based typing methods focus on exon 2 and exon 3 for HLA class I loci and exon 2 alone for HLA class II loci because the regions are most polymorphic and encode the peptide-binding groove that binds to HLA antigens. Therefore, we further trimmed a range of the genomic sequences that contain corresponding exon(s) as the target sequences (the short rectangle in the bottom). There are alleles that are identical over exons 2 + 3 for HLA class I and exon 2 for HLA class II. To avoid ambiguity, we selected one allele from each group of alleles with identical target sequences. Table 2 lists the reference alleles, the starting positions and lengths of the trimmed range on the reference genomic sequences, and the numbers of alleles with unique target sequences for loci HLA-A, B and DRB1.

**Table 2:** **Three HLA loci and their corresponding reference alleles**

	A	B	DRB1
Reference	A*01:01:01:01	B*07:02:01	DRB1*01:01:01
Start	380	400	5400
Length	1100	950	600
#unique alleles	2335	3075	1388

#### Reads generated from the target sequences

The CCS reads for the target sequence of an allele were produced with PBSIM [21], which is the only simulator that generates PacBio libraries as far as we know. We adopted its model-based method and the default settings for CCS reads (length-mean=450, length-sd=170, accuracy-mean=0.98, accuracy-sd=0.02).

#### Types of runs

To estimate the number of reads required to genotype, we designed three types of runs, all of which contain the same number of reads and different numbers of samples (see Table 3). Since the number of barcode pairs for multiplex sequencing is 48 [11], the number of samples in a run is a factor of 48. In each run, we set the total number of reads for a locus (amplicon) as 960 because the number of usable CCS reads is about 15 K for 1 Kb insert and multiple loci of HLA are sequenced simultaneously. We simulated ten groups of samples for each type of runs. For each group of samples, we re-generated the reads and applied our method ten times.

**Table 3:** **Three types of runs with the same total number of reads: 960**

	Type 1	Type 2	Type 3
#correct reads/allele	40	20	10
#samples/group	12	24	48
#groups	10	10	10

#### Noise reads

To understand the impacts of barcode-calling errors, in each run and for each locus, we created a pool of reads that contained the reads of the locus from all the samples in the run. Before genotyping a sample, a few number of reads were randomly selected from the pool to disturb correct reads (i.e. the reads generated from the sample). We call such reads as noise reads. Five different levels of noise reads (0, 10, 20, 30, 40 noise reads) were added.

**Figure 2:**
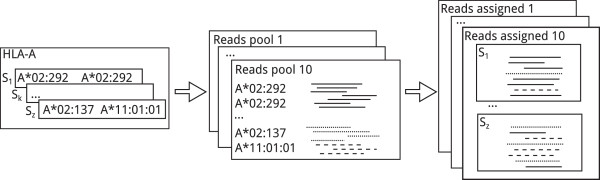
**Simulating pools of reads for a group of samples.** Left: simulating the alleles of the samples. Middle: 10 iterations of generating the reads. Right: mixing reads assigned to correct samples with noise reads.

Figure 2 gives an illustration of the simulation process.

### Bayes’ theorem for HLA typing

Given the set of reads assigned to a sample (see the smaller rectangles in the rightmost block of Figure 2), we used Bayes’ theorem to infer the pair of alleles (*a*_*p*_,*a*_*q*_) of the sample.

Denote the reads as *r*_1_,*r*_2_,…,*r*_*n*_ and a pair of alleles as *a*_*i*_, *a*_*j*_.
1

The probability *p*(*a*_*i*_,*a*_*j*_), which is the probability of a random sample having the allele pair (*a*_*i*_,*a*_*j*_), depends on the population of the sample and we assumed all *p*(*a*_*i*_,*a*_*j*_)’s are the same when the population is unknown. To find the pair of alleles (*a*_*p*_,*a*_*q*_) that maximize formula (1) when the *r*_1_,…,*r*_*n*_ are fixed, it is sufficient to compare *p*(*r*_1_,…,*r*_*n*_|*a*_*i*_,*a*_*j*_).

Given the alleles that produce the reads, the reads are independent of each other. Therefore,
2

With the alleles *a*_*i*_ and *a*_*j*_, a read can be produced from only one of them. Therefore, we set the probability of a read given the pair of alleles to be the the higher probability of the read given only one of the pair of alleles.
3

Equations 2 and 3 lead to
4

Denote the error rate of sequencing as *δ*, the number of matches of the alignment between *r*_*k*_ and *a*_*i*_ as |*r*_*k*_=*a*_*i*_|, the number of mismatches as |*r*_*k*_≠*a*_*i*_| and the length of *r*_*k*_ as |*r*_*k*_|.
5

Under this definition, *p*(*r*_*k*_|*a*_*i*_) stands for the probability when a sequence of length |*r*_*k*_| generated by *a*_*i*_ equals the sequence of *r*_*k*_. The summation of this function for all possible sequences of length |*r*_*k*_| would be 1 and therefore, it is a legal probability function.

Since it remains the same to compare log*p*(*r*_1_,…,*r*_*n*_|*a*_*i*_,*a*_*j*_) instead of *p*(*r*_1_,…,*r*_*n*_|*a*_*i*_,*a*_*j*_), we have
6

Using this method, the number of reads produced by *a*_*p*_ (or *a*_*q*_) can be estimated as the number of reads whose |*r*_*k*_=*a*_*p*_| is greater than |*r*_*k*_=*a*_*q*_|. When the number of reads of one allele is far less than that of the other (e.g. 50%), the sample is regarded as having two identical alleles.

This method works well when the given set of reads are all from the alleles of the sample (i.e. the correct reads). However, the barcode-calling errors might result in mixing reads from different samples (i.e., the noise reads). Alleles that are close to both the correct reads and the noise reads are more likely to be predicted as the answers. To deal with the problem, we assumed there are a few number of noise reads before selecting the pair (*a*_*p*_,*a*_*q*_).

Denote *m* as the ratio of noise reads assumed. We select the pair
7

In the equation, *ρ*_*k*_=0 means the read *r*_*k*_ is a correct read and *ρ*_*k*_=1 means *r*_*k*_ is a noise read. Note that equation 7 is the same as equation 6 when *m*=0. We name the methods based on equation 6 and equation 7 as *BayesTyping*0 and *BayesTyping*1, respectively.

The value |*r*_*k*_=*a*_*i*_| can be calculated by aligning the read *r*_*k*_ and the allele *a*_*i*_. We use the score of the alignment instead of the number of matches in our program because the score catches more information (e.g. indels).

### Implementation

**Figure 3:**
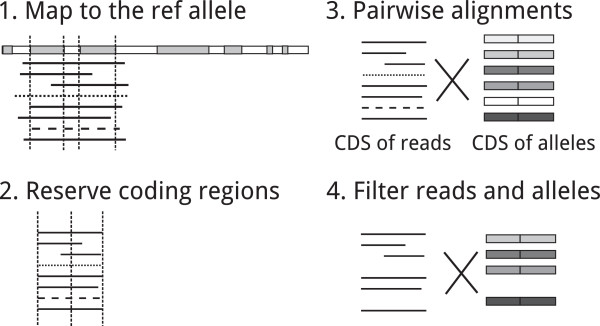
**Pre-processing steps of our methods.** **1)**. Map the reads to the reference genomic sequence. **2)**. For each read, trim its introns by reserving the sequences mapping to the coding regions of the reference. **3)**. Align each reserved sequence to the CDS of each allele respectively. **4)**. Filter unlikely reads and alleles.

Given the set of reads assigned to a sample, we used LASTZ [22] to map the reads to the genomic sequence of the reference allele (identity=90, coverage=70). For each read, we trimmed the regions aligned to introns and only reserved those regions aligned to the exons of the genomic sequence (see Figure 3-2). The reads that had short sequences left (less than 50 bp) were eliminated. We then used LASTZ to map the remained reads to the coding sequences of all the unique alleles (explained in Section 'Methods’) separately. We chose a lower gap penalty and a low gap extension penalty (100, 20) since PacBio reads tend to have more indels. The alignment results of all pairs of the remained reads and the unique alleles were saved and only the best score for each (read, allele) pair was maintained.

To accelerate, we excluded those unlikely alleles, which had less than ten percent of reads satisfying |*r*_*k*_=*a*_*i*_|= maxall *x*|*r*_*k*_=*a*_*x*_| because we assume the correct alleles tend to have more number of best matching reads. To reduce those impacts of noise reads, we excluded the unlikely reads, which had less than 96% of identities to all the remained alleles. The pre-processing steps are illustrated in Figure 3.

At last, we used the methods *BayesTyping*0 and *BayesTyping*1 to infer the pair of alleles and their corresponding reads. The original sequences of the corresponding reads can further be used to assemble the genomic sequences of the alleles.

## Results and discussion

We compared the first method BayesTyping0 with NGSengine, which is a platform-independent software for NGS data analysis of HLA genes. The number of reads for a sample in Type 2 and Type 3 experiments seems too few for NGSengine and it could not predict any alleles. The Type 1 experiments contain 1,200 sets of reads (12 samples/group × 10 groups × 10 iterations) and each set contains 80 reads (40 correct reads/allele × 2 alleles). We regarded a successful prediction when the two predicted alleles are both correct. Without inducing noise reads, when typing HLA-A, NGSengine could only successfully predicted 274 pairs of alleles (22.83%). On the other hand, BayesTyping0 successfully predicted 1199 pairs of alleles (99.92%). NGSengine requires more reads to achieve the same accuracy (data not shown). We listed the accuracies of BayesTyping0 for the three HLA loci and the three types of experiments without noise reads in Table 4.

**Table 4:** **Accuracies of BayesTyping0 for experiments without noise reads**

	HLA-A	HLA-B	HLA-DRB1
Type 1	99.92%	99.92%	100.00%
Type 2	99.50%	99.21%	100.00%
Type 3	97.62%	96.87%	99.98%

The three types of runs are as defined in Table 3.

For experiments with noise reads, we compared our methods with a method *MaxTwo*, which gives the first two alleles by comparing the number of reads having the maximum alignment scores with them. For all the three methods, when the number of reads of one allele is less than 50% of the number of the other allele, the sample is regarded as having two identical alleles.

**Figure 4:**
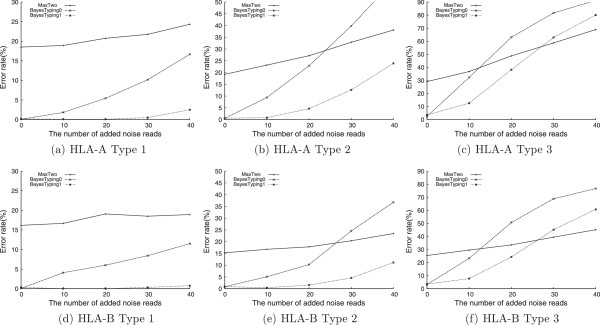
**Experiments results of HLA-A and HLA-B genotyping.** We plotted the results of the three types of runs for HLA-A and HLA-B genotyping in **(a)**-**(c)** and **(d)**-**(f)** respectively, using three different methods. The x-axis stands for the numbers of added noise reads and y-axis stands for the error rates.

We repeated the experiments by adding different numbers of noise reads. We set the assumed ratio of noise reads *m* for BayesTyping1 as 20% of the number of input reads (correct reads + noise reads). Figure 4 shows the error rates for HLA-A and HLA-B, respectively. For HLA-DRB1, all the three methods could identify the correct alleles quite well. The accuracy is about 99% even for the type 3 experiments with the most number of noise reads (20 correct reads and 40 noises reads).

It could be presumed that the error rates would increase as the number of noise reads increases, e.g., when there are more barcode-calling errors, or the number of correct reads decrease, e.g., when multiplexing more samples. For example, for HLA-A, the error rate is more than 10% for type 3 experiments when only 10 noise reads are induced. BayesTyping1 showed the best capability to tolerate the disturbance of the noise reads. Even when there were no noise reads, which conflicted with the assumption of BayesTyping1, BayesTyping1 also performed well. One the other hand, BayesTyping0 usually performed best when there are no noise reads, but it suffered as the number of noise reads increased. It even performed worse than MaxTwo when the noise reads outnumbered the correct reads.

The difference error rates between the three loci might reflect the characteristics of the sequences of alleles currently gathered. Although the number of HLA-B alleles is more than that of HLA-A, the HLA-B sequences seem more distinguishable because the error rates of typing were lower. It seems that HLA-DRB1 has the best distinguishable alleles.

**Figure 5:**
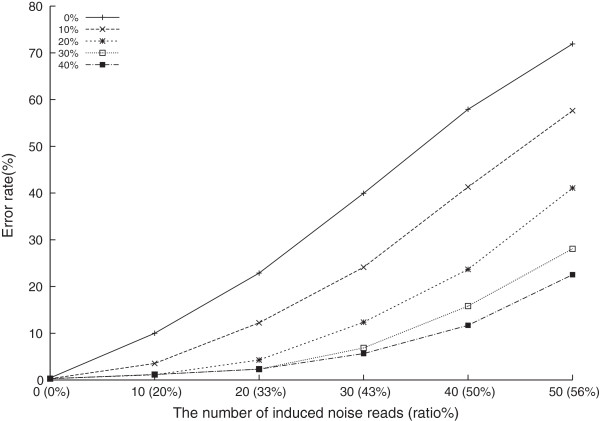
**The change of error rates with different levels of noise reads.** Experiments of Type 2 HLA-A using BayesTyping1 with different levels of *m*, which is the ratio of noise reads assumed.

To study the effect of the parameter *m* for BayesTyping1, we set *m* as different percentages of the number of the input reads and ran BayesTyping1 repeatedly. The data we used are Type 2 HLA-A experiments and more noise reads were added. We plotted the results in Figure 5.

It showed that a higher level of *m* worked better when noise reads were added, i.e., given any vertical line, a higher level of *m* has a fewer error rate. The difference of error rates also becomes larger as the number of noise reads increases. However, the error rates would converge at some point and a larger *m* would make little effect, i.e., given any vertical line, the difference of error rates shrinks as the level of *m* increases. When *m* is too large and there are only few numbers of noise reads, BayesTyping1 will perform worse than BayesTyping0 (data not shown). Theoretically, all pairs of alleles have the same probability when *m* is 100% of the input reads.

### Ambiguous allele combinations

For data with read length not long enough (such as 450 bp in the simulation), there are still ambiguous allele combination problems to type class I HLA. Assuming there are four alleles with the following patterns of exons,


When the read length is not long enough to cover the region of exon 2 + intron + exon 3, for the samples with allele a and allele b (or allele c and allele d), there is no way to distinguish which combination of alleles is correct.

To address this problem, we enumerated such ambiguous pairs of alleles for HLA-A and randomly selected 24 pairs for the samples in a Type 2 run. The 24 ambiguous pairs of alleles were listed in Additional file 1: Table S2. Except that the average read length was 1 Kb, other steps and parameters to generate the reads were exactly the same as described in Simulation. To make a contract, we also generated reads with average length 450 bp and doubled the number of reads for each allele (from 20 to 40) to reach similar depth of coverage. As in Experiments, we also re-generated both types of reads ten times for this group of samples. Different levels of noise reads were induced and we applied BayesTyping1 to genotype. The results are summarized in Table 5. In addition to the number of times that BayesTyping1 correctly assigned the pairs of alleles, we also listed the number of times when BayesTyping1 had more than one answers (the correct answer was included).

**Table 5:** **The results of BayesTyping1 on 1 Kb and 450 bp reads for 24 samples with ambiguous pairs of alleles**

#Noise reads	0	10	20	30	40
1 Kb (correct)	240	240	240	240	240
1 Kb (ambiguous)	0	0	0	1	1
450 bp (correct)	234	231	235	229	212
450 bp (ambiguous)	132	69	34	7	5

It showed that using 1 Kb reads, BayesTyping1 could correctly assign the pairs of alleles without ambiguity in most cases, even when the number of the noise reads equalled the number of correct reads. On the other hand, using 450 bp reads, BayesTyping1 could also achieve good accuracies. This might be due to the variation of PacBio read length. With higher depth of coverage, there are still a few reads that are long enough to cover exon 2 and exon 3. It is surprising that BayesTyping1 caused much more ambiguities when the number of noise reads was fewer. It might be because BayesTyping1 tends to treat longer reads as noise reads when there are no noise reads in fact.

### Diversity of noise reads

The source of noise reads might also affect the error rates of typing. In the worst case, when the noise reads are all from an allele of another sample, it is more likely to identify the wrong allele. When the noise reads are diverse, the corrected alleles might be easier to stand out.

**Figure 6:**
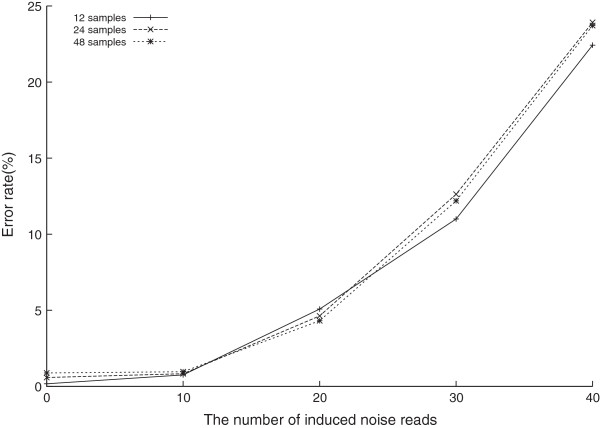
**The influence of the diversity of the read pools.** Experiments of HLA-A with noises from different numbers of samples using BayesTyping1.

To compare the impact of the diversity of the noise reads, we mixed noise reads from read pools that contained different number of samples, i.e. 12, 24 and 48, respectively, with correct reads. Other parameters are the same as Type 2 experiments. The experimental results using HLA-A were shown in Figure 6.

The error rates of these three experiments showed not much difference. The number of correct reads, the number of noise reads and the difference between the two numbers play a much more important role.

### Homozygous and heterozygous samples

As mentioned in Section 'Methods’, for each loci, we simulated 30% of homozygous samples. To test whether the accuracies of homozygous samples and those of heterozygous samples are significantly different or not, we utilized Fisher’s exact test [23]. We summed the numbers of correct and wrong predictions for homozygous and heterozygous samples of the HLA-A and HLA-B experiments. (HLA-DRB1 experiments were excluded since most of the predictions are correct.) The contingency table of the four number were expressed in Table 6. The tables of the three types of runs can be found in Additional file 1: Table S3-S5. For each type of experiments, the total sum should be {(#loci) × (#samples) × (#groups of samples) × (#iterations) × (#levels of noise reads)} (2×24×10×10×5 for the type 2 experiments).

**Table 6:** **Four numbers for Fisher’s exact test of type 2 experiments**

	Homozygous	Heterozygous
Correct	6720	15814
Error	330	1136

We applied Fisher’s exact test for the contingency tables of the three types of experiments and listed the p-values and odd ratios in Table 7. The odds ratio is calculated by {(#correct for homozygous)/(#error for homozygous)}/{(#correct for heterozygous)/(#error for heterozygous)}. It showed homozygous samples had more advantages over heterozygous samples when the number of correct reads were fewer. It might be because the number of correct reads for the same allele doubled for homozygous samples, which made the correct allele stand out.

**Table 7:** **Results of Fisher’s exact test**

	Type 1	Type 2	Type 3
#reads/allele	40	20	10
Odds ratio	0.66	1.46	1.47
p-value	0.13	1.2×10^-9^	2.2×10^-16^

## Conclusions

The experimental results showed that BayesTyping1 can identify HLA alleles accurately using reasonably low number of PacBio CCS reads. BayesTyping1 can tolerate sequencing errors, which are introduced by the PacBio sequencing technology, and noise reads, which are introduced by barcode-calling errors, to some degree. The three types of experiments suggest it is better to multiplex 12 or 24 samples instead of 48 samples to maintain a high accuracy, since the number of reads for each sample in a 48-sample example might be too few for HLA typing.

## Electronic supplementary material

Additional file 1: **It contains the table listing the frequencies of the 2-digits alleles of the three loci: HLA-A, HLA-B and HLA-DRB1.** It also contains the twenty-four pairs of ambiguous alleles we used in our experiment and the contingency tables for Fish’s exact test. (PDF 81 KB)

## References

[1] Mallal S, Nolan D, Witt C, Masel G, Martin A, Moore C, Sayer D, Castley A, Mamotte C, Maxwell D, James I, Christiansen FT (2002). **Association between presence of HLA-B* 5701, HLA-DR7, and HLA-DQ3 and hypersensitivity to HIV-1 reverse-transcriptase inhibitor abacavir**. The Lancet.

[2] Lie BA, Thorsby E (2005). **Several genes in the extended human MHC contribute to predisposition to autoimmune diseases**. Curr Opin Immunol.

[3] Tiercy J-M (2002). **Molecular basis of HLA polymorphism: implications in clinical transplantation**. Transpl Immunol.

[4] Tait BD (2011). **The ever-expanding list of HLA alleles: changing HLA nomenclature and its relevance to clinical transplantation**. Transplant Rev.

[5] Gao X, Bashirova A, Iversen AK, Phair J, Goedert JJ, Buchbinder S, Hoots K, Vlahov D, Altfeld M, O’Brien SJ, Carrington M (2005). **AIDS restriction HLA allotypes target distinct intervals of HIV-1 pathogenesis**. Nat Med.

[6] Robinson J, Halliwell JA, McWilliam H, Lopez R, Parham P, Marsh SG (2013). **The IMGT/HLA database**. Nucleic Acids Res.

[7] Erlich H, Opelz G, Hansen J (2001). **HLA DNA typing and transplantation**. Immunity.

[8] Lee S. J, Klein J, Haagenson M, Baxter-Lowe LA, Confer DL, Eapen M, Fernandez-Vina M, Flomenberg N, Horowitz M, Hurley CK, Noreen H, Oudshoorn M, Petersdorf E, Setterholm M, Spellman S, Weisdorf D, Williams TM, Anasetti C (2007). **High-resolution donor-recipient HLA matching contributes to the success of unrelated donor marrow transplantation**. Blood.

[9] Middleton D (1998). **History of DNA typing for the human MHC**. Rev Immunogenet.

[10] De Santis D, Dinauer D, Duke J, Erlich H, Holcomb C, Lind C, Mackiewicz K, Monos D, Moudgil A, Norman P, Parham P, Sasson A (2013). **Allcock RJ: 16th ihiw: Review of hla typing by ngs**. Int J Immunogenet.

[11] PacBio: **Multiplexing Targeted Sequencing using Barcodes****.** Technical Note PN 100–114–500–01, Pacific Biosciences 2012, [http://www.pacificbiosciences.com/pdf/TN_Multiplexing_Targeted_Sequencing_Using_Barcodes.pdf]

[12] Adams SD, Barracchini KC, Chen D, Robbins F, Wang L, Larsen P, Luhm R, Stroncek DF (2004). **Ambiguous allele combinations in HLA Class I and Class II sequence-based typing: when precise nucleotide sequencing leads to imprecise allele identification**. J Transl Med.

[13] Roberts RJ, Carneiro MO, Schatz MC (2013). **The advantages of SMRT sequencing**. Genome Biol.

[14] PacBio: **Targeted Sequencing – SNP Detection and Validation****.** Technical Note PN 100–092–600–03, Pacific Biosciences 2012,

[15] Huang X, Madan A (1999). **CAP3: A DNA sequence assembly program**. Genome Res.

[16] Huang X, Wang J, Aluru S, Yang S-P, Hillier L (2003). **PCAP: a whole-genome assembly program**. Genome Res.

[17] Shaw CK, Chen LL, Lee A, Lee TD (1999). **Distribution of HLA gene and haplotype frequencies in Taiwan: a comparative study among Min-nan, Hakka, Aborigines and mainland Chinese**. Tissue Antigens.

[18] **The Allele Frequency Net Database** [http://www.allelefrequencies.net]

[19] Gonzalez-Galarza FF, Christmas S, Middleton D, Jones AR (2011). **Allele frequency net: a database and online repository for immune gene frequencies in worldwide populations**. Nucleic Acids Res.

[20] **IGMT/HLA Database** [http://www.ebi.ac.uk/ipd/imgt/hla/]

[21] Ono Y, Asai K, Hamada M (2013). **PBSIM: PacBio reads simulator–toward accurate genome assembly**. Bioinformatics.

[22] Harris RS: **Improved pairwise alignment of genomic dna****.** *PhD thesis*. The Pennsylvania State University; 2007

[23] Agresti A (2002). Categorical Data Analysis, vol 359.

