# Supplementary material for: A fault-tolerant method for HLA typing with PacBio data
Source: BMC Bioinformatics. 2014 Sep 3;15(1):296. doi: 10.1186/1471-2105-15-296 (PMC4161847; doi:10.1186/1471-2105-15-296)
Supplement: Supplementary file 1 — Additional file 1: It contains the table listing the frequencies of the 2-digits alleles of the three loci: HLA-A, HLA-B and HLA-DRB1. It also contains the twenty-four pairs of ambiguous alleles we used in our experiment and the contingency tables for Fish’s exact test. (PDF 81 KB) [file 12859_2014_6558_MOESM1_ESM.pdf]

A Fault-tolerant Method for HLA Typing with PacBio Data

Chia-Jung Chang[1], Pei-Lung Chen[2,3,4,5], Wei-Shiung Yang[2,3,4], and Kun-Mao Chao[1,6]

[1] Department of Computer Science and Information Engineering, National Taiwan University, No.1, Sec.4, Roosevelt Road, Taipei, Taiwan (R.O.C.), 10617.
[2] Departments of Medical Genetics and Internal Medicine, National Taiwan University Hospital, No. 8, Chung Shan S. Road, Taipei, Taiwan (R.O.C.), 10041.
[3] Graduate Institute of Clinical Medicine, National Taiwan University College of Medicine, No. 1, Sec. 1, Jen Ai Road, Taipei, Taiwan (R.O.C.), 10051.
[4] Research Center for Developmental Biology and Regenerative Medicine, National Taiwan University, No.1, Sec. 4, Roosevelt Road, Taipei, Taiwan (R.O.C), 10617.
[5] Graduate Institute of Medical Genomics and Proteomics, National Taiwan University, No.1, Sec.4, Roosevelt Road, Taipei, Taiwan (R.O.C.), 10617.
[6] Graduate Institute of Biomedical Electronics and Bioinformatics, National Taiwan University, No.1, Sec.4, Roosevelt Road, Taipei, Taiwan (R.O.C.), 10617.

Additional file 1

Table S1. Three types of experiments with the same number of reads

A		B		DRB1	
Name	Freq (%)	Name	Freq (%)	Name	Freq (%)
A*01	0.30	B*07	0.30	DRB1*01	0.30
A*02	31.70	B*08	0.10	DRB1*03	8.40
A*03	0.30	B*13	8.30	DRB1*04	14.80
A*11	34.90	B*14	0.10	DRB1*07	2.20
A*23	0.10	B*15	13.10	DRB1*08	9.10
A*24	16.60	B*18	0.20	DRB1*09	15.40
A*25	0.10	B*27	2.70	DRB1*10	0.50
A*26	1.30	B*35	1.50	DRB1*11	8.00
A*29	0.10	B*37	0.20	DRB1*12	14.10
A*30	1.20	B*38	3.70	DRB1*13	3.70
A*31	1.20	B*39	2.60	DRB1*14	6.40
A*32	0.20	B*40	24.20	DRB1*15	9.90
A*33	11.60	B*41	0.10	DRB1*16	3.90
A*34	0.10	B*42	0.10		
A*36	0.10	B*44	0.50		
A*43	0.10	B*45	0.10		
A*66	0.10	B*46	14.60		
A*68	0.10	B*47	0.10		
A*69	0.10	B*48	1.20		
A*74	0.10	B*49	0.10		
A*80	0.10	B*50	0.10		
		B*51	6.00		
		B*52	0.80		
		B*53	0.30		
		B*54	2.50		
		B*55	3.00		
		B*56	0.80		
		B*57	0.80		
		B*58	10.70		
		B*59	0.10		
		B*67	0.10		
		B*73	0.10		
		B*78	0.10		
		B*81	0.10		
		B*82	0.10		
		B*83	0.10		

Table S2. Twenty four samples with ambiguous alleles.

allele 1	allele 2
A*02:01:01:01	A*02:54
A*02:37	A*02:06:01
A*02:01:22	A*02:474
A*02:07:01	A*02:06:04
A*23:01:13	A*24:156
A*24:02:58	A*23:51
A*02:127	A*02:364
A*02:170	A*02:167
A*02:64	A*02:170
A*02:364	A*02:178
A*11:16	A*11:116
A*11:140	A*11:73
A*02:01:22	A*02:300
A*02:110	A*02:06:04
A*02:12	A*02:404
A*02:423	A*02:142
A*02:03:01	A*02:438
A*02:190	A*02:355
A*31:01:05	A*33:18:01
A*31:06	A*33:03:05
A*02:40:01	A*02:438
A*02:190	A*02:51
A*01:26	A*11:39
A*01:136	A*11:94

Table S3. The contingency table for Type 1 experiments

	homozygous	heterozygous
#correct	4075	7868
#error	25	32

Table S4. The contingency table for Type 2 experiments

	homozygous	heterozygous
#correct	6720	15814
#error	330	1136

Table S5. The contingency table for Type 3 experiments

	homozygous	heterozygous
#correct	9950	21798
#error	3850	12402
